# Impact of small-scale vegetation structure on tephra layer preservation

**DOI:** 10.1038/srep37260

**Published:** 2016-11-15

**Authors:** Nick A. Cutler, Olivia M. Shears, Richard T. Streeter, Andrew J. Dugmore

**Affiliations:** 1 Scott Polar Research Institute, University of Cambridge, UK; 2 Department of Geography, University of Cambridge, UK; 3 Department of Geography & Sustainable Development, University of St Andrews, UK; 4 School of Geosciences, University of Edinburgh, UK

## Abstract

The factors that influence tephra layer taphonomy are poorly understood, but vegetation cover is likely to play a role in the preservation of terrestrial tephra deposits. The impact of vegetation on tephra layer preservation is important because: 1) the morphology of tephra layers could record key characteristics of past land surfaces and 2) vegetation-driven variability in tephra thickness could affect attempts to infer eruption and dispersion parameters. We investigated small- (metre-) scale interactions between vegetation and a thin (<10 cm), recent tephra layer. We conducted surveys of vegetation structure and tephra thickness at two locations which received a similar tephra deposit, but had contrasting vegetation cover (moss vs shrub). The tephra layer was thicker and less variable under shrub cover. Vegetation structure and layer thickness were correlated on the moss site but not under shrub cover, where the canopy reduced the influence of understory vegetation on layer morphology. Our results show that vegetation structure can influence tephra layer thickness on both small and medium (site) scales. These findings suggest that some tephra layers may carry a signal of past vegetation cover. They also have implications for the sampling effort required to reliably estimate the parameters of initial deposits.

Layers of tephra (pyroclastic fragments of different sizes) preserved in the stratigraphic record are sometimes uniform, with sharply defined upper and lower boundaries. However, these layers often exhibit a remarkable degree of small-scale variation in thickness and/or diffuse contacts with surrounding sediments, even when the tephra layer in question is of similar age and thickness to a uniform deposit (refer to Supplementary Fig. S1 for examples). Variations in tephra layer thickness reflect both volcanic processes and post-depositional changes that take place as tephra are stabilised and incorporated into the sedimentary section. Recent work has suggested that vegetation cover at the site of deposition may influence the morphology of thin, terrestrial tephra layers on a landscape scale^1^ (smaller-scale relationships remain largely unexplored). If vegetation structure does influence preservation, then some tephra layers could, in principle, be used to infer characteristics of the surface environment contemporaneous with the eruption. Whether that is the case or not, it is still essential to understand the extent to which a tephra layer within the stratigraphic record is representative of the initial fallout, as layer thickness is frequently used to reconstruct the volcanic plume that led to the deposit. Our study is a systematic assessment of the relationship between vegetation structure and the morphology of tephra layers at metre and sub-metre scales. As such, we explore the potential of terrestrial tephra layers as a source of environmental data and the impact that vegetation cover can have on attempts to infer eruption and dispersion parameters from these deposits.

There are two broad types of explanation for the thickness variations observed in terrestrial tephra layers. Firstly, the variations may be a result of post-depositional modifications of the tephra. These may be driven by processes such as cryoturbation^2^, solifluction^3^ and biological processes such as animal burrowing^4^. Secondly, variations in thickness may be a result of the characteristics of the surface the tephra falls upon. This could be as a result of the initial deposit falling on snow or ice and subsequently being moved around as the snow melts^4^,^5^. Alternatively, land surface characteristics, particularly vegetation structures, may alter how tephra are retained and preserved through their effect on the wind field^6^, especially if the deposit is relatively thin (1–10 cm). There may also be linkages between pre- and post-depositional processes. For example, re-mobilised tephra are likely to be preferentially retained in areas that can trap more of the initial fallout. While there has been significant progress on understanding the impact of vegetation cover on geomorphological processes^7^,^8^,^9^,^10^, tephra deposits may behave differently because they are generally a short-lived, ballistic input of sediment rather than semi-continuous input.

Recent research has demonstrated that vegetation structure can influence mean tephra layer thickness on a landscape scale^1^,^11^. Given that most land surfaces have some sort of vegetation cover, a greater understanding of the interaction between plants and tephra deposits would have global utility in the interpretation of tephra layers and their origins. A number of researchers have sought to understand the spatial variability of cryptotephra deposits in peatlands^12^,^13^,^14^,^15^,^16^, but this work has mainly focussed on distinguishing primary tephra from reworked deposits, and has not considered variations in the thickness of visible deposits. With a few notable exceptions^17^, small- (metre and sub-metre-) scale relationships between vegetation structure and tephra layer morphology remain unexplored. If plants do influence tephra layer preservation, then patchiness in vegetation structure should produce patchiness in tephra layer thickness. By extension, if a recent, relatively thin, tephra layer was to be examined and it was certain the that the extant vegetation cover resembled that at the time of deposition, vegetation structure and tephra layer thickness would be expected to co-vary in space (i.e. the scales of patchiness in the vegetation and tephra layer should be similar). Correlation between small-scale vegetation structure and tephra layer thickness could give new insights into land surfaces at the time of past volcanic eruptions. In addition, the signal generated by heterogeneous vegetation could have a major impact on the production of isopach maps from stratigraphic records and hence the reconstruction of past fallout volumes.

To assess this phenomenon, we surveyed a recently deposited tephra layer in areas of contrasting vegetation cover. We focussed on the fate of a thin (<10 cm) deposit produced during the eruption of the Grímsvötn volcano in 2011 (hereafter referred to as the G2011 tephra). We chose two locations in Fossdalur, Iceland, where the vegetation persisted in an unchanged state after tephra deposition. The proximity of the sites meant that they received a similar thickness of compositionally-identical tephra during the G2011 eruption. The sites represented opposite ends of a continuum of vegetation structure (Fig. 1). The Fossdalur-moss site (Fm) was mossy heathland; the Fossdalur-birch (Fb) site had a canopy of short-statured (generally < 4 m tall) birch and a grassy ground layer. We conducted high-resolution (centimetre-scale) surveys of tephra thickness and vegetation structure (based a metric of vegetation height (U0.7^1^) and % canopy cover on the Fb site) along 20 m long transects. We anticipated that:

1. Mean tephra layer thickness would be higher in the taller vegetation;
2. Both vegetation structure and tephra thickness would be positively autocorrelated (patchy) at a metre scale;
3. The scale of patchiness in the vegetation structure would match that in the tephra layer.

We also calculated the variance in tephra thickness, to estimate the minimum number of samples required to reliably estimate mean thickness in each vegetation type.

## Results

### Vegetation

The Fm site was dominated by the moss *Racomitrium lanuginosum*. The *Racomitrium* was mixed with other moss species (*R. ericoides* and *Sanionia uncinata*) to form a dense ground layer a few centimetres thick. Graminoids were also present: *Kobrobesia myosuroides* and *Festuca* sp. were common; *Trisetum spicatum* (a rush) and sedges (*Carex* spp.) were abundant but scattered. Low-growing forbs typical of upland heath in Iceland (e.g. *Thymus praecox, Galium verum, Bistorta vivipara*) were also observed, along with horsetails (*Equisetum* spp.)

The Fb site was characterised by continuous downy birch (*Betula pubescens*) scrub. Individual trees were around 1.5–4.0 m high. Shorter shrubs (e.g. *Salix phylicifolia, Vaccinium uliginosum* and *Rubus saxatilis*) occurred where the birch canopy was thinnest. Mean canopy density was 63 ± 0.6% and only varied within a narrow range (~55–70%). The ground layer was up to ~15 cm tall (but mostly much lower) and dominated by grasses, mainly *Festuca* sp., with sparse patches of sweet vernal grass (*Anthoxanthum odoratum*). There were also scattered forbs, notably *Geranium sylvaticum* and *Angelica sylvestris* and isolated patches of moss (*Hylocomium splendens*) forming small (<10 cm high) hummocks.

Ground layer vegetation was significantly taller on the Fb site (t = 21.4, p < 0.001). The mean value of U0.7 on the Fb site was 0.056 ± 0.001 m, as opposed to 0.027 ± 0.001 m on the Fm site (Fig. 2). Variability in vegetation height was similar: the coefficient of variance (CV) for both the Fb and Fm sites was 43%.

### Tephra

The G2011 tephra was significantly thicker on the Fb site (t = 23.2, p < 0.001) (Fig. 2). The mean thickness was 0.052 ± 0.0004 m on the Fb site, compared with 0.036 ± 0.0006 m on Fm. Unlike the vegetation height measurements, tephra layer thickness was considerably more variable under the mossy vegetation: the CV for Fm was 32.4%, compared with 16.8% under the birch. The difference in variance between the two sites meant that the number of samples required to accurately establish mean thickness was different. On the Fb site, 31 samples would be required to establish the mean to within 5%, with a confidence level of 95%. On the Fm site, the equivalent figure was 114 samples.

Scatterplots of vegetation height and tephra thickness with distance illustrate these differences. The birch canopy on Fb was relatively open (mean canopy density = 63%) but more-or-less continuous. The proportion of cover varied in a narrow range (SD = 4.6%) and there were no obvious gaps in tree cover (Fig. 3a). Canopy density was not correlated with ground layer height (Pearson product-moment: r = −0.16, p = 0.24). Both sites had peaks and troughs in vegetation height (Fig. 3b,c). However, it was clear that whilst tephra thickness on the Fb site was more-or-less constant along the transect, the tephra layer on Fm was uneven and displayed a humped relationship with distance (Fig. 3d,e). Vegetation height and tephra thickness were positively correlated on Fm (Pearson product-moment: r = 0.37, p < 0.001) but not on Fb (r = −0.06, p = 0.26) (Fig. 3f,g). Furthermore, tephra thickness on Fb was not correlated with canopy density (Pearson product-moment: r = 0.1, p = 0.44).

### Spatial autocorrelation

The correlograms of vegetation height and G2011 thickness on Fm exhibited pattern typical of patchy vegetation, i.e. high, positive autocorrelation at short distances, with a declining trend at longer lags (Fig. 4a). Vegetation height was positively autocorrelated up to ~4–5 m, indicating relatively large patches of a similar height (Fig. 4c). Negative correlation at long lags was generated by the comparison of dissimilar patches (i.e. short and tall vegetation). Tephra thickness generated a similar correlogram, with positive autocorrelation up to a lag of ~3 m. Given similarities in the scales of patchiness, it was not surprising that the cross-correlogram exhibited positive autocorrelation over a range of few metres (Fig. 4e).

The presence of spatial structure was much less apparent on Fb. Weak, positive autocorrelation was present in vegetation height, but only at the shortest lag distance (~0.5 m: Fig. 4b). Similarly, short-range autocorrelation was observed in tephra, but the autocorrelations, both positive and negative, were weak (Fig. 4d). The cross-correlogram showed negative autocorrelation at short lags (up to ~1 m), indicating that patchiness in the ground layer vegetation and tephra layer was out of phase (Fig. 4f).

The analysis of canopy density indicated very strong positive autocorrelation up to ~1.5 m. However, the cross-correlograms with tephra thickness showed no obvious covariance in the two variables along the Fb transect (Fig. 4g).

## Discussion

Random effects operating at the scale of individual plants might be expected to obscure the relationship between vegetation structure and tephra layer thickness at (sub-)metre-scales. However, our study showed that under specific circumstances small-scale variability in vegetation structure can leave a legacy in the morphology of tephra layers. This relationship was demonstrated at the Fm site, where the vegetation was relatively simple and there was only one structural layer. On this site, measurements of vegetation height at 5 cm intervals were positively correlated with tephra thickness at the same interval. The two variables were patchy at similar scales (~3–5 m) and covaried in space at short lags (Fig. 4e).

These observations have important implications for environmental reconstruction, because they show that tephra layers may contain useful data on past vegetation structure (information which may be difficult, if not impossible, to obtain any other way). If this is the case, tephra layers could give insight into ecological resilience at the time of deposition, as subtle changes in vegetation structure have been demonstrated to precede abrupt state changes, i.e. they may be considered early warning signals of ecological thresholds^18^,^19^,^20^. Data from the Fm site support previous research that suggested tephra layers may record ecological resilience at the time of deposition. Streeter and Dugmore surveyed the G2011 tephra in eroded and vegetated areas either side of an active erosion front and observed marked differences in the variability of layer thickness^21^. They interpreted these differences in tephra layer morphology as indicators of ecological resilience and posited that as an external driver (e.g. increased grazing pressure or climate change) pushes an ecosystem towards an abrupt change of state, changes in vegetation height and stem density occurred. These differences were then reflected in the morphology of a tephra deposit.

The tephra layer at the wooded Fb site was significantly thicker than the layer from the non-wooded, heathland site (Fm). As both sites received a similar initial tephra deposit and only differed in terms of vegetation cover, this result suggested enhanced tephra retention within woodland. This finding supports the conclusions of Cutler *et al*.^1^ who also found correlations between vegetation structure and tephra thickness at a landscape scale. We argue that two factors contributed to enhanced preservation under the birch canopy: a) a reduction in wind velocity created by the birch trees and b) the existence of a regional mosaic of vegetation types, among which tephra retention varied (i.e., remobilised tephra was intercepted by the patches of birch woodland).

Correlations between ground layer vegetation structure and tephra thickness were not apparent on the wooded Fb site. The Fb ground layer was just as variable as that on non-wooded site, but the tephra layer under the birch was remarkably homogeneous. Both vegetation height and tephra layer thickness were autocorrelated over very short distances (~0.5 m), but the strength of the relationship was very weak: the shape of the correlograms was consistent with low levels of variability (Fig. 4b,d). Cross-correlation of vegetation height and tephra thickness was negative at short lags (i.e. the thickest tephra was associated with the shortest vegetation) although this relationship was again very weak and not related to obvious patterns in the data.

Tephra thickness on Fb was not correlated with canopy structure, either, although the variation in canopy density was small. The correlogram of canopy density suggested patchiness on a scale < 2 m, but this is to be expected, given the inevitable overlap between the canopy images. Overall, the impression gained from the survey was that the birch canopy on the Fb site was spatially homogeneous, with few significant openings (none in our survey). Our results suggested that the birch canopy decoupled the tephra deposit from the ground layer.

Based on the data in this paper and the findings of Cutler *et al*.^1^, we propose the following conceptual model of small-scale tephra-vegetation interactions in temperate, humid climates. In open, short-statured vegetation, exposed conditions result in losses of fine tephra from the land surface and a thin tephra layer. Even small variations in vegetation height/density impact on tephra retention and, subsequently, tephra layer morphology (Fig. 5a). When scattered shrubs are present, a wind environment with sharp local variations is created. This leads to the thickening of tephra layers under shrub patches and marked small-scale variability in layer thickness (Fig. 5b). In addition to aeolian processes, the differential impact of rain-splash under variable canopy cover may influence tephra layer morphology at metre-scales^11^.

A shrub or tree canopy is likely to intercept a portion of the airfall deposit^22^. Some of the trapped material may be subsequently lost from the system by, e.g. wind. However, it is likely that the majority is transferred to the ground over a period of days-weeks^23^. Where the shrub canopy is more-or-less continuous (as it was on the Fb site), it will ‘sift’ tephra-fall to smear out small-scale variation. Wind speeds under the canopy are low, so small variations in ground layer structure are less significant than they would be in exposed locations. Tephra that percolates through the canopy mantles the woodland floor and is likely to be retained *in situ*, regardless of variability in the ground layer (Fig. 5c).

In a spatially patchy canopy, greater variability in tephra thickness might be expected. For example, in a forest with large, well-spaced trees, variations in tephra thickness caused by the shedding of material trapped in the canopy are possible. For example, local thickening around the perimeter of each tree crown was observed in coniferous forests after the eruption of Mount St Helens in 1980, probably due to the shedding of trapped tephra^24^. Furthermore, in a canopy with obvious gaps, a negative correlation between canopy density and tephra thickness might be expected, as taller ground layer vegetation in clearings could become influential.

If the influence of vegetation on tephra preservation at small spatial scales can be quantified, then it is possible that tephra layers could be used as more than just correlational or dating horizons or a means of inferring past volcanic activity. They could also be used as a means of inferring patchiness in vegetation cover from the time of the eruption, with possible implications for the inference of long-term changes in vegetation structure and resilience. This approach could complement other methods for understanding vegetation cover such as palynology, which provide a detailed understanding of the how regional plant species composition changes through time, but are less useful as a proxy for vegetation structure. It may be especially useful where the same species can have very different growth forms, e.g., *Betula* spp., which range from 0.5 m tall shrubs to 20 m tall trees, according to conditions.

The findings reported here could find applications within archaeology, e.g. understanding patterns of forest clearance. In Iceland forest clearance proceeded swiftly after the late Ninth Century Norse introduction of grazing mammals and a pastoral subsistence system^25^,^26^. Clearance developed over several centuries^27^ as cleared zones extended from settlement sites. The detailed spatial patterns of this change are currently unknown, but could be mapped using thickness variations of the Katla (c. 920 CE) and Eldgja (c. 938 CE) tephra deposits, which cover extensive areas of southern Iceland in layers 1–10 cm thick.

There are clear implications for the reconstruction of palaeo-eruptions from relatively thin tephra layers, should relationships between vegetation structure and tephra thickness apply more generally. If the fallout from an eruption is deposited on spatially heterogeneous vegetation, then the resulting tephra layer would be expected to vary according ground cover, as well as distance from the eruption. This would have a particularly marked effect on the reconstruction of eruptions where the fallout spans ecotones. This commonly occurs in regions such as the southern Andes^28^, where topographically-induced climate variation leads to major changes in vegetation over comparatively short distances. In such cases, accurately inferring initial deposit thickness would be dependent upon knowledge of contemporary vegetation structure.

There are also implications for field surveys of tephra layer thickness. Although a few high-resolution measurements of tephra layer morphology have been taken before^21^, to our knowledge, this is the first tephra layer survey to make measurements at centimetre intervals over such long distances (tens of metres). Given the large number of samples collected, we are confident that our estimates of mean thickness closely approximate the true mean. Clearly, such a large number of samples would not be required in most surveys. However, the results clearly illustrate how a single tephra layer can vary in space and this has implications for efforts to reconstruct initial deposits. Even in the least variable scenario (on the Fb site, where the birch canopy appears to have smoothed-out variations in thickness), >30 samples would be required to estimate mean thickness reliably. In the adjacent mossy site, >100 samples would be required, even though the vegetation looks relatively homogeneous to the naked eye. In spatially heterogeneous vegetation (e.g. shrub patches in a matrix of shorter vegetation), variability in tephra thickness is likely to be very high, with correspondingly higher uncertainty. It would be impractical to collect such large numbers of samples when reconstructing palaeo-eruptions. However, an appreciation of the impact of vegetation cover on the preservation of tephra should lead to more efficient sampling strategies as well as better estimates of error ranges.

Our study was conducted on a relatively small spatial scale, without site-level replication. Consequently, we were unable to demonstrate a causal link between vegetation structure and tephra layer morphology. Further replicated studies, preferably in other volcanic environments, would put our findings on a firmer footing. The experimental application of tephra to contrasting vegetation types, coupled with repeated longitudinal measurements of tephra layer thickness, would also be helpful in developing a mechanistic understanding of tephra layer taphonomy. Such experimental approaches have been successfully applied in understanding tephra layer formation on peatlands^13^ and on bioturbation of tephra in marine environments^16^. Studies of plant macrofossils might improve understanding of vegetation-tephra interactions in older deposits. Finally, numerical modelling exercises (possibly based on pre-existing models of aeolian deposition) might extend knowledge of tephra preservation on a landscape scale.

## Conclusions

We demonstrated a positive correlation between vegetation structure and the thickness of tephra deposits and showed that, in certain circumstances, small-scale variations in vegetation structure may be reflected in tephra layers. If this relationship between vegetation structure and tephra preservation applies more generally, our work has wide-ranging implications for environmental reconstruction, archaeology and the inference of eruption parameters from relatively thin (1–10 cm) tephra layers. This is significant, because tephra layers of this type may be spatially extensive (up to thousands of km^2^) and can account for the bulk of tephra-fall deposits from large, explosive eruptions^29^,^30^. Our findings have implications for the sampling effort required to reliably estimate deposit parameters: thin tephra layers formed on short, structurally heterogeneous vegetation will demand more samples. Our study indicates that small-scale (metre and sub-metre) relationships can emerge in short-statured, structurally simple vegetation, where variations in vegetation height/density modify local wind fields and influence tephra retention. The presence of a closed shrub or tree canopy is likely to reduce surface wind speeds in an even way, allowing tephra to mantle the ground surface. This decouples the eventual morphology of the tephra layer from the ground layer, resulting in a spatially homogeneous deposit.

## Methods

### Sampling locations

**Figure 1:**
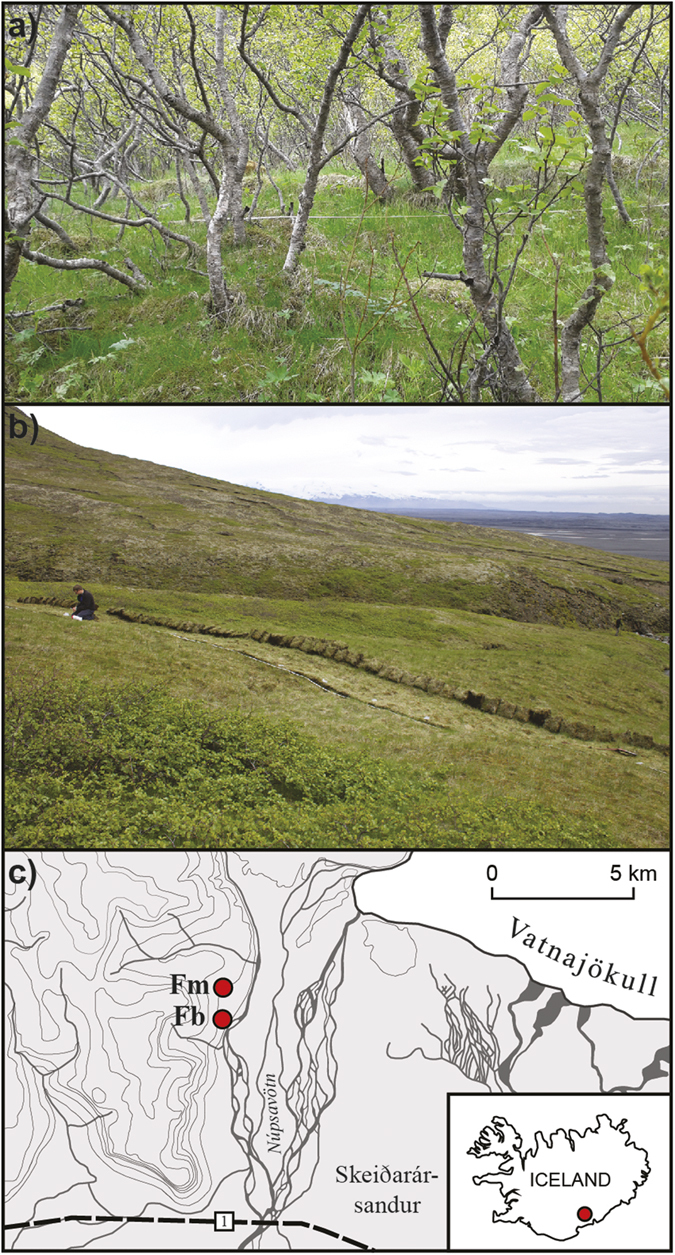
The survey locations (**a**) Fossdalur birch (Fb); (**b**) Fossdalur moss (Fm); (**c**) location plan. Figure 1c was drafted in Adobe Illustrator CS5 (www.adobe.com/illustrator), using publicly available cartographic data supplied by the National Land Survey of Iceland (www.lmi.is/en).

**Figure 2:**
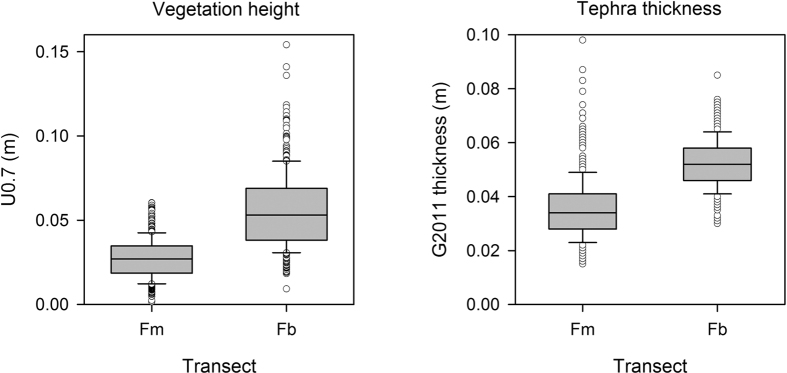
Vegetation height (left) and tephra thickness measurements for each transect.

**Figure 3:**
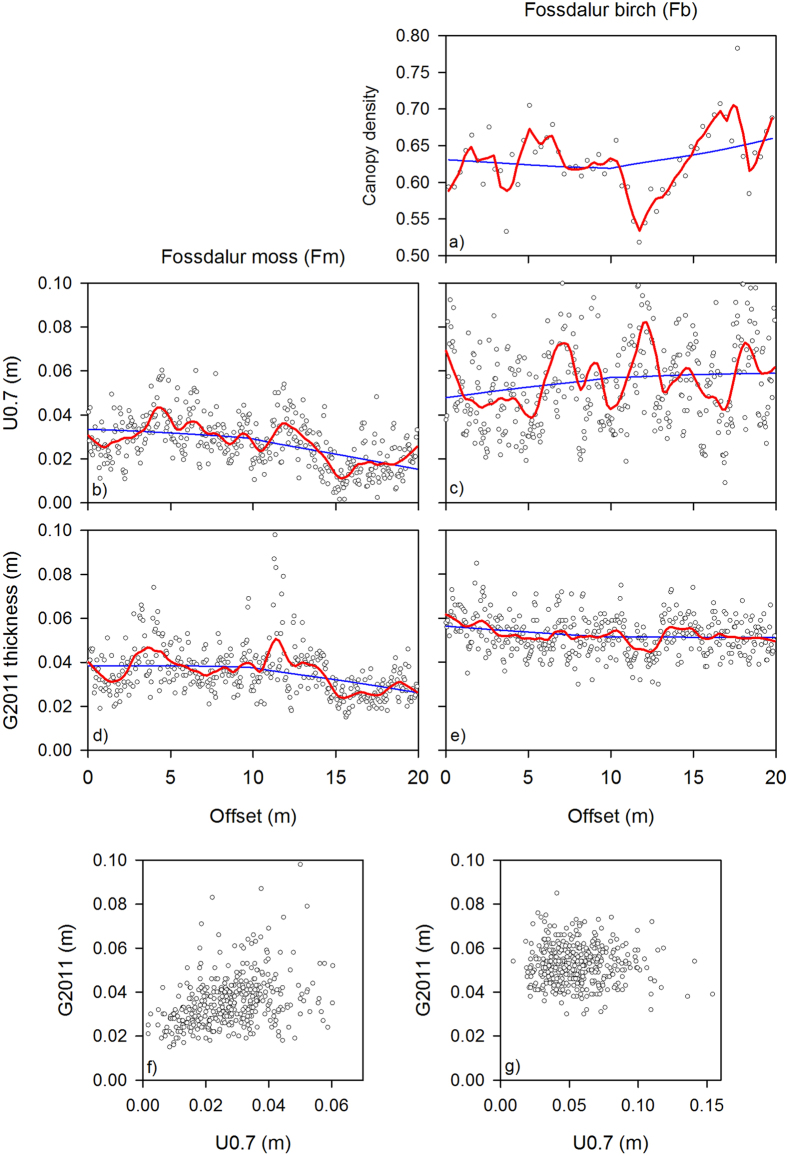
Variations in canopy density on Fb (**a**); vegetation height (**b,c**) and tephra depth (**d,e**) along the two transects. The scatterplots at the bottom of the figure show the relationship between the two variables on Fm and Fb, respectively (**f,g**). The lines in figs a-e are LOESS smoothers; the blue line is based on the whole dataset; the red line has a smoothing parameter of 0.1.

**Figure 4:**
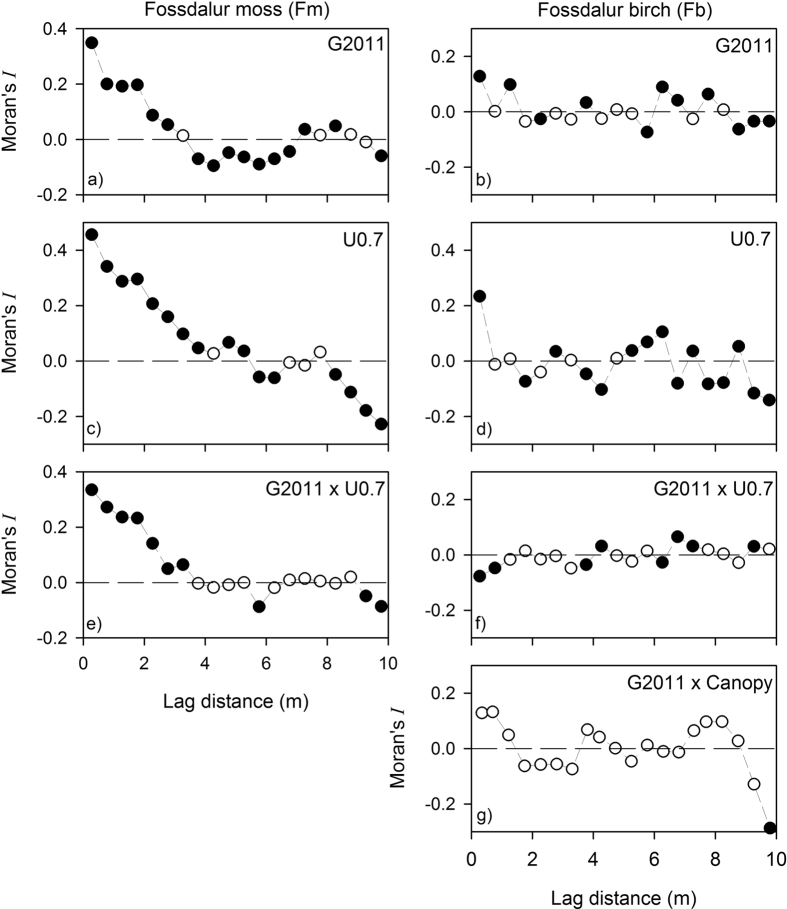
Correlograms for tephra thickness (**a,b**) and vegetation height (**c,d**) for the two transects; cross-correlograms of tephra thickness and vegetation height (**e,f**) and tephra thickness and canopy density (**g**), the latter for Fb only. Filled points indicate significant autocorrelation (p < 0.05).

**Figure 5:**
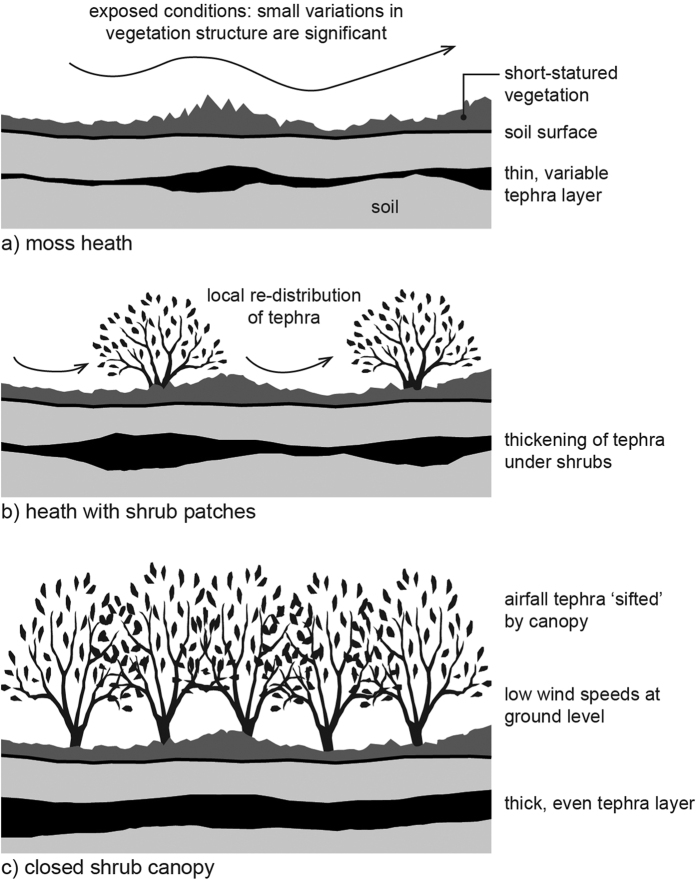
A conceptual model illustrating the impact of varying vegetation cover on a tephra layer resulting from the same initial deposit (**a**) in open, short-statured vegetation; (**b**) a ground layer with scattered shrubs; (**c**) a continuous shrub canopy.

We conducted high-resolution surveys of vegetation structure and tephra thickness at two locations in Fossdalur, Iceland in June 2015 (Fig. 1). The sampling locations had contrasting vegetation cover, representing end members of the vegetation cover present in Iceland. One site (Fossdalur moss, Fm: 63° 59′ 41.3″ N, 17° 29′ 3.1″ W) was mossy heathland, the other (Fossdalur birch, Fb: 64° 0′ 1.7″ N, 17° 29′ 0.8″ W) had a canopy of birch and a grassy ground layer (Fig. 1a,b). The sites are in an area that received tephra fallout during the eruption of Grímsvötn in 2011 (G2011), in a location where the thinning rate of the fallout was low^31^ and post-depositional disturbance was unlikely. We selected the sites ‘blind’ (i.e. without conducting preliminary surveys), based on vegetation cover, landscape characteristics (similarity in altitude, aspect and surface morphology) and accessibility. Because of their close proximity (the sites are a few hundred metres apart) and the fact they lie on the main axis of fallout from the eruption, we assumed that the initial tephra deposit was similar in both locations^32^. We also assumed that the plants we surveyed were representative of vegetation cover at the time of the eruption as the event was a) recent and b) comparatively minor, in terms of thickness of the initial layer (~5 cm). Icelandic plant communities change slowly because of their depauperate and eurytopic character, combined with consistency of climate and land use. The resilience of the plant communities means that they are generally unaffected by the deposition of a few centimetres of tephra^33^,^34^.

### Data acquisition

**Figure 6:**
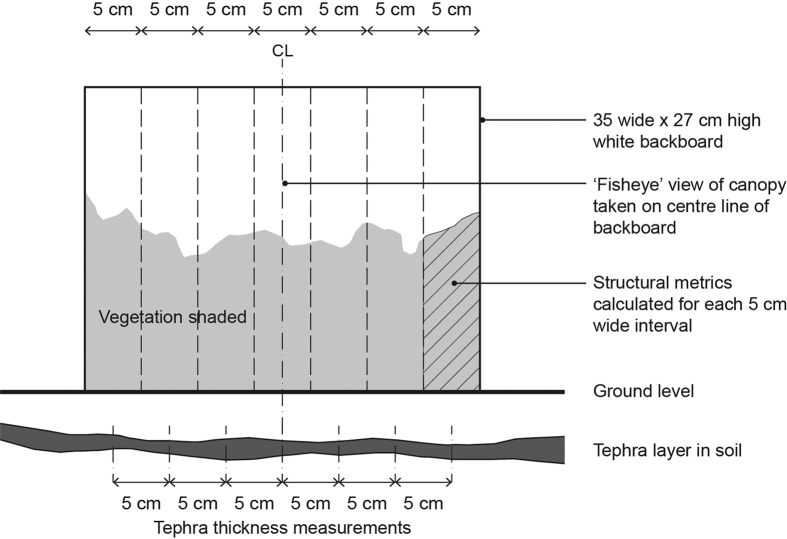
Surveying tephra thickness and vegetation along transects. Ground layer vegetation structure was recorded with side-on photographs, using a 35 × 27 cm backboard as a reference (57 contiguous images/transect). The image was subsequently divided into seven 5 cm wide sections. Measurements of tephra thickness were made at 5 cm intervals, corresponding to the centre point of each 5 cm slice of vegetation. A upward facing image of the birch canopy was taking from ground level on the Fb transect, with the camera positioned on the centre line of the backboard.

We used 20 m long transects to survey G2011 tephra thickness and vegetation structure in both locations. A shallow (~10 cm deep) trench was excavated along the transect line to reveal the G2011 tephra layer. The layer was readily identified as it formed a distinct, dark layer in the upper horizon of the soil. The thickness of the tephra was measured to the nearest millimetre at 5 cm intervals (399 values per transect). The dominant plant species in the ground cover were established by visual inspection and identified following the taxonomy of Kristinsson^35^. Vegetation structure was recorded photogrammetrically, following a methodology developed by Zehm *et al*.^36^. Briefly, contiguous, side-on digital photographs were taken of the vegetation adjacent to the transect, using a 35 cm long x 27 cm high backing board for scale calibration (57 photographs per transect). The images were processed so that ground-layer vegetation was rendered as black pixels and the background as white pixels. The black pixels were then enumerated using a bespoke routine written in MATLAB, allowing metrics of vegetation density and height to be derived. Based on previous studies, we used a metric of vegetation height as an explanatory variable (specifically, the height below which 70% of vegetation occurred, termed U0.7 hereafter). This metric is not as sensitive to outliers (e.g. slim, individual stems) as maximum vegetation height, and provides a robust indicator of vegetation structure^1^. By dividing each image into seven 5 cm sections, we were able to calculate vegetation height at intervals which corresponded to the tephra measurements (Fig. 6).

Clearly, side-on photography can only capture the structure of ground layer vegetation. In of order to quantify canopy density on the Fb site, we took upward-facing photographs from the woodland floor, using a fisheye lens^37^. Photographs were taken at 35 cm intervals, corresponding to the centre points of the side-on photographs (Fig. 6). The digital camera used was levelled using a spirit level (to ensure that the film plane was horizontal) and operated with a remote shutter release. The images were then rendered in a similar way to the side-on photographs, such that branches and leaves were represented by black pixels and the sky as white. The proportion of black pixels was then calculated as a metric of canopy density and compared to tephra thickness averaged over a 35 cm interval. The survey was conducted in June 2015, when the birch trees were in full leaf (the G2011 eruption occurred in late May). Weather conditions in early 2015 were unseasonably cold^38^ and vegetation growth was somewhat retarded. We therefore assumed that the plants we observed were at a similar developmental stage to those present at the time of the eruption.

### Analysis

Mean values for tephra thickness and vegetation height were compared with t-tests, assuming uneven variance. Coefficients of variance (CV) were also calculated. The large number of samples collected gave us a good estimate of population variance for tephra thickness. We were therefore able to estimate the sampling effort required to reliably estimate mean tephra thickness to an arbitrary level (in this case, we estimated the number of samples required to estimate mean thickness to within 5%, with a 95% confidence level). We did this using equation 1, following Eckblad^39^:

Number of samples ≅ [(coefficient of variation * t-value)/accuracy]^2^ (equation 1)

Where the t-value has N-1 (=398) degrees of freedom and a p-value < 0.05; accuracy = 0.05.

The spatial structure of the tephra layers and vegetation was described using correlograms (for a single variable) and cross-correlograms (for comparing the covariance of G2011 thickness and vegetation height)^40^. The range of small-scale, positive autocorrelation allows the typical scale of patchiness in a dataset to be estimated^40^ (refer to Supplementary Fig. S2 for further details). If G2011 thickness and vegetation height are related, one would expect autocorrelation at similar spatial scales. The degree of autocorrelation was calculated according to Moran’s *I*, which usually varies between 1 (perfect, positive autocorrelation) and −1 (perfect, negative autocorrelation). Moran’s *I* is sensitive to outliers, so the tephra layer data were checked for normality before the analysis. Only lags up to half the total transect length were considered as the number of comparisons at the longest separation distances is necessarily small. The significance of the calculated values of *I* was estimated by permutation tests (99 randomisations), using Holm’s correction method to account for multiple testing. The analysis was implemented using the ncf package running in R^41^.

## Additional Information

**How to cite this article**: Cutler, N. A. *et al*. Impact of small-scale vegetation structure on tephra layer preservation. *Sci. Rep.* **6**, 37260; doi: 10.1038/srep37260 (2016).

**Publisher’s note:** Springer Nature remains neutral with regard to jurisdictional claims in published maps and institutional affiliations.

## Supplementary Material

Supplementary Information
