# Supplementary material for: Impact of small-scale vegetation structure on tephra layer preservation
Source: Sci Rep. 2016 Nov 15;6:37260. doi: 10.1038/srep37260 (PMC5109036; doi:10.1038/srep37260)
Supplement: Supplementary Information [file srep37260-s1.pdf]

Impact of small-scale vegetation structure on tephra layer preservation

Nick A Cutler, Olivia M Shears, Richard T Streeter and Andrew J Dugmore

Supplementary Information

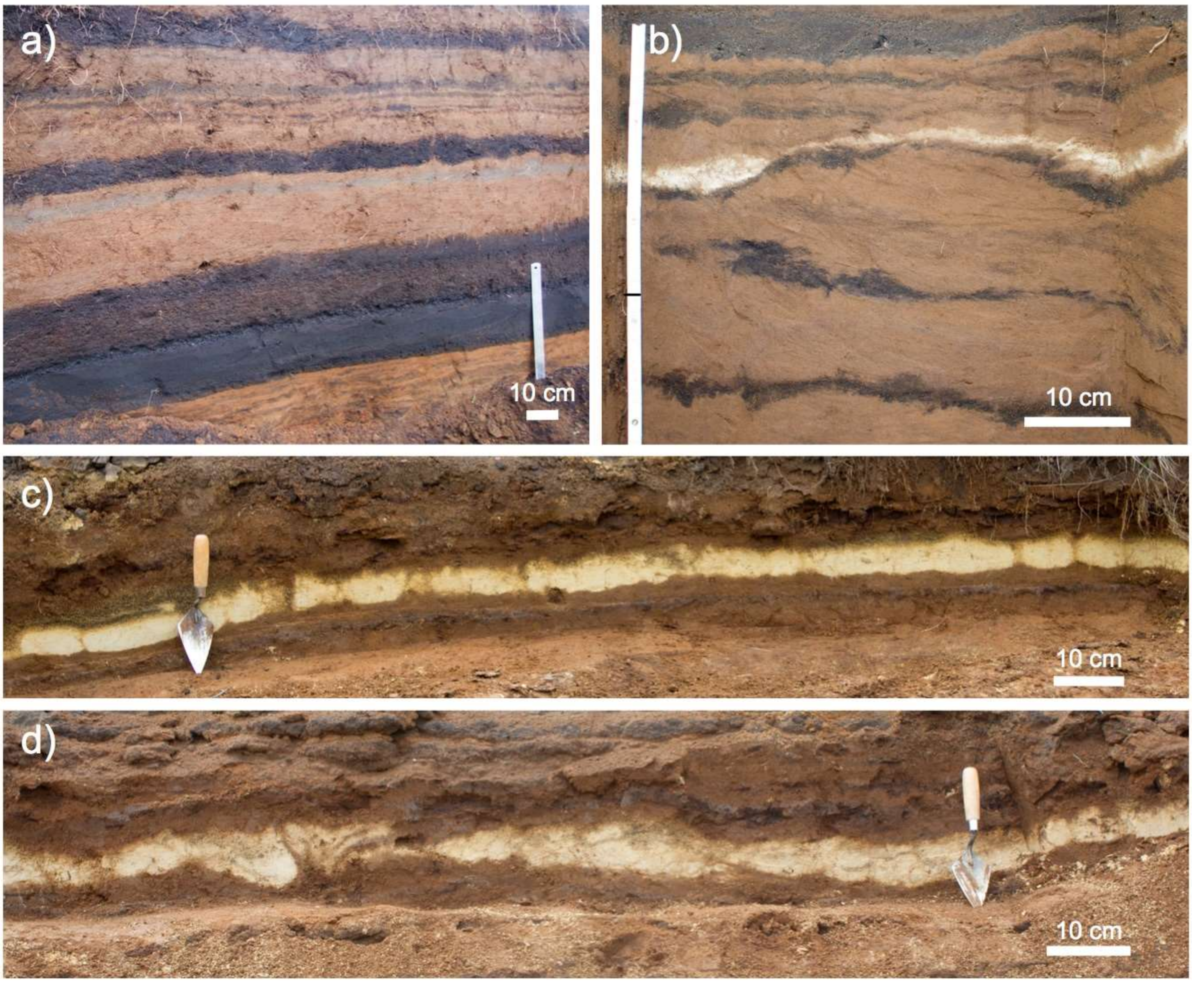

Supplementary fig. S1.

Sub-metre-scale variability in tephra layers, illustrated by Icelandic stratigraphic sections: a) a section in Skaftártunga which shows tephra layers of varying thickness but low variability (the thick black layer at the bottom of the section is from the Eldgjá eruption in the early 10th century CE and the uppermost dark tephra is Katla 1625 CE); b) highly variable, thin tephra layers at Kalfafell, southern Iceland (the prominent, light coloured tephra is from the eruption of Öræfjajökull in 1362 CE, the dark coloured layers are from Katla and Grímsvötn); c) low thickness variability observed in the HS Hekla tephra (3515±55 BCE) at Kalfafell; d) a variable H4 (Hekla) tephra (3826±12 BCE), also from Kalfafell: the structures on the left hand side of the image might represent shrub stems at the time of the eruption.

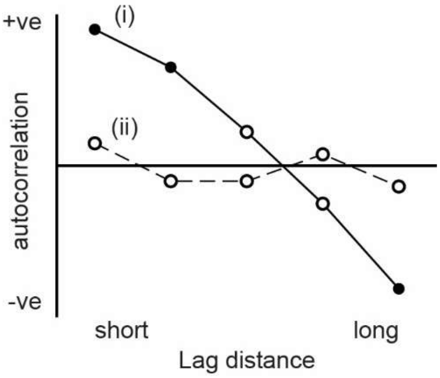

Supplementary Fig. S2
Hypothetical correlograms illustrating two different relationships between separation distance (lag) and autocorrelation. The separation distance between pairs of points increases from left to right; filled points exhibit significant autocorrelation. The profile labelled (i) would be characteristic of a spatially patchy phenomenon: closely-spaced samples are similar and positively autocorrelated. As the separation distance increases, so does the degree of positive autocorrelation. Negative autocorrelation occurs when widely-spaced points in dissimilar patches are compared. The profile labelled (ii) would indicate complete spatial randomness as autocorrelation is close to zero at all lags.
